# Editorial: Measuring diets and food choice in the context of a changing world

**DOI:** 10.3389/fnut.2025.1571706

**Published:** 2025-06-11

**Authors:** Shauna Downs, Winnie Bell, Christine E. Blake

**Affiliations:** ^1^Department of Health Behavior, Society, and Policy, School of Public Health, Rutgers, The State University of New Jersey, Piscataway, NJ, United States; ^2^Intake - Center for Dietary Assessment at FHI 360, Washington, DC, United States; ^3^Department of Health Promotion, Education, and Behavior, Arnold School of Public Health, University of South Carolina, Columbia, SC, United States

**Keywords:** food choice, diet quality, food environment, diets, diet measurement

Rapid changes to the food environments that consumers interact with, and the broader food system drivers that influence them, have led to a nutrition transition that is impacting the health of people and the planet (1–3). Global trends in the prevalence of underweight and obesity point to a worsening of malnutrition in all its forms in most countries (4), the world is off track to meet most of the global nutrition goals (5), and poor-quality diets remain a leading cause of disease worldwide (6). To begin addressing these issues, increased collection of representative quantitative dietary intake data—particularly in low- and middle-income countries—is necessary. However, this is not sufficient (7, 8); there is also a clear need for innovative approaches to improving food choices and diets that are grounded in the realities of a changing world that is grappling with conflict, climate variability and change, political instability, and the strong influence of corporate interests, among others.

As incomes rise and local food environments undergo rapid changes providing consumers with a wider array of options at competitive prices, understanding the underlying drivers of food choice including the preferences and values that underpin them has become increasingly important (9, 10). In recent years there have been renewed efforts to understand the drivers of food choice in the context of changing food environments and broader food systems, and how these drivers link with food consumption (11, 12). The goal of this Research Topic was to look beyond dietary consumption (what people eat) by considering more holistically the various drivers of food choice (how and why people eat the foods they do), to better understand what underpins individual decisions (13). Ultimately, by understanding why people make the choices they do—especially when situated in the local food environment and/or broader food system—we can identify important entry points for improving diets, reducing malnutrition, and strengthening food systems to better support food choice.

This Research Topic comprises 15 manuscripts, including perspectives, reviews, and original research undertaken in Europe, North America, Latin America, West Africa, Southeast Asia, and the Pacific Islands. The manuscripts contribute to our knowledge of the role of food environments in influencing food access, the factors that influence food choices in diverse contexts, including new ways of measuring those factors, how to capture and analyze dietary data in innovative, and less labor-intensive ways, as well as insights into ways to intervene within food systems to improve food choices and health outcomes.

We know that the food environments that people get food from, as well as the characteristics of those environments such as food availability, affordability, promotion, etc., interact with personal factors to influence the foods that consumers eventually acquire, purchase, and consume (14). Four papers in the Research Topic explore the importance of different food environment types on promoting food access. The articles by Coffin-Schmitt et al., Downs et al., and Zeitler et al. examine the importance of the natural food environment (where people access wild and cultivated foods) in supporting food security across diverse contexts including upstate New York, the Mekong River region of Cambodia, and among indigenous populations in Thailand. Together these papers provide us with a better understanding of where people access their food from, what motivates them to access food from these spaces, and how their decisions change across seasons or in the face of climate, economic, or other shocks, such as the COVID-19 pandemic. While accessing food from the natural food environment can be an important coping strategy to help address food insecurity (15), as countries move through the stages of the nutrition transition, there tends to be a shift away from the natural food environments toward more built environments (3, 16). Domínguez-Barreto et al. explored the shift in built food environments from more informal to formal food outlets that has coincided with a reduction in the reliance on public markets in Mexico between 1994 and 2020. These changes in how consumers engage with their food environments have implications in terms of food purchasing and consumption, particularly as it relates to the consumption of fresh foods compared to ultra-processed foods.

Consumers' lived experiences accessing food are influenced by their food environments as well as the personal factors or drivers that they value and consider when making decisions about which foods to consume. Five papers in the Research Topic examine drivers of food choices among Italian, Fijian, Senegalese, and American populations. Carfora and Catellani examined psychosocial drivers of local food purchasing among Italian consumers finding that the availability of local food was the main driver of its purchase along with other important factors such as trust in local food producers, authenticity, taste, social sustainability such as workers' rights, and appearance. Boxer et al. conducted a rapid review to examine factors influencing dietary behaviors in Fiji, finding that individual preferences for processed foods (particularly among younger populations) and gender and social dynamics that favored meat and overconsumption (particularly among men) were influencing food choices, despite knowledge about what constitutes a healthy diet. Other key drivers of dietary behaviors included food safety and climate variability, the latter of which created difficulties in planting and growing crops and disrupted supply chains leading to an increased reliance on processed and packaged foods. In recognition of the important role women play in influencing food choices within families, Hamam et al. aimed to examine the relationships between involvement in food choices and eating patterns. Through cluster analysis, they identified four types of women's eating behaviors, including: hedonic food consumers, sustainable- and balanced-diet consumers, food experimenters, and consumers with no food fondness. Vanderkooy et al. assessed the relationship between unhealthy food and beverage consumption with diet quality among 12–35 month children in Senegal finding that children who consumed diets high in unhealthy food and beverages had lower micronutrient intakes, tended to be older, and were more likely to be food insecure than those with lower intakes. They also explored the drivers of commercial processed food and beverages which included child preferences, the use of these products as behavioral management tools, or as treats and gifts. These findings can help provide insight into behavior change communication strategies that promote nutrient-rich foods that children enjoy in lieu of commercial processed foods. Gutjahr et al. designed and validated a dietary protein assessment questionnaire to explore US college students' knowledge and attitudes toward dietary protein. The final questionnaire includes questions that assess views on the relationships between meat production and consumption and the environment and the importance of organic sources of protein in terms of the health of people and the environment. With further testing and validation, this tool could potentially be used to create more effective nutrition interventions for college students.

We know that capturing dietary data is labor intensive and it requires skills and training to collect it in a way that generates high quality data (17). One of the contributions that we set out to make with this Research Topic was to identify novel methods for collecting and analyzing data that can then be used to inform what people are eating, and what motivates people to eat (or not eat) the foods they do. Both Gligorić et al. and Schäufele-Elbers and Janssen leverage routinely collected consumer sales and purchase data to measure food choices with a particular focus on sustainability, the latter of which examined the gaps between intentions and real-world food purchasing in the context of sustainability. Treitler et al. used citizen science (i.e., when members of the general public participate in the scientific process) with adolescents in Virginia, USA, and compared it with the National Health and Nutrition Examination Survey data, to assess adolescent nutrient intakes, demonstrating the potential for novel citizen science approaches to provide insight into diet quality. Di Maso et al. adapted the Nutritional Functional Diversity indicator, derived by ecologists, to examine dietary diversity finding that it was strongly associated with common dietary quality scores, specifically the Mediterranean Diet Score (18) and the Healthy Eating Index (19). These papers provide insight into novel methods for both collecting and analyzing dietary data in ways that can help inform which aspects of the diet need to be targeted in interventions, programs, or policies as well as the evaluation of the impact of those strategies on dietary outcomes.

One of the main reasons for striving to better understand the food environments that consumers are exposed to, and how the drivers of food choice interact with them to influence food choices, is to better inform the development of interventions, programs, and policies aimed at shifting consumers toward higher quality diets and improved nutrition and health outcomes. Brooker et al. reviewed the existing evidence of the effectiveness of interventions implemented in food retail settings and found that pricing strategies had the most favorable impacts on food purchasing outcomes, particularly among rural populations and those with low socio-economic status. They also found that promotional strategies combined with other initiatives were effective among the general population. It is likely that drawing on the evidence related to the drivers of food choice within specific populations can help tailor interventions such as the ones examined in the article by Brooker et al. and lead to larger improvements in food choice. In addition to interventions that are prevention-oriented and implemented at the population level, as countries undergo the nutrition transition, those interventions will need to be complemented with programs targeting individuals with existing diet-related non-communicable diseases, such as diabetes. However, there are often resource and capacity constraints that create barriers to reaching people in need (20). As part of this Research Topic, Chisaguano-Tonato et al. helped to address this gap by creating an Ecuadorian food exchange list comprised of 404 foods to assist health professionals and researchers streamline the process of meal planning in an efficient and effective way that is aligned with cultural practices and habits.

While continued efforts are needed to increase the collection of representative quantitative dietary intake data—particularly in low- and middle-income countries—the papers included in this Research Topic demonstrate the importance of going beyond what people eat and exploring the underlying drivers and contexts that influence food choice to better design interventions that are tailored to people's lived experiences. It is likely that many of the interventions that will be truly impactful in terms of shifting the burden of poor-quality diets and malnutrition in all its forms will need to be context-specific and co-created in a way that centers people's lived experiences. As we move toward this type of approach, innovative methods, including leveraging citizen science, will have an integral role to play in designing and evaluating these types of food systems actions.
